# A Social Norms and Identity Approach to Increasing Fruit and Vegetable Intake of Undergraduate Students in the United Kingdom

**DOI:** 10.3389/fpsyg.2022.838394

**Published:** 2022-05-17

**Authors:** Wanda Fischera, Mara van Beusekom, Suzanne Higgs, Joanne E. Cecil

**Affiliations:** ^1^School of Medicine, North Haugh, University of St Andrews, St Andrews, United Kingdom; ^2^School of Psychology, College of Life and Environmental Sciences, University of Birmingham, Birmingham, United Kingdom

**Keywords:** social norms, descriptive norm, fruit, vegetable, identification, eating behavior

## Abstract

This study investigated the influence of descriptive norm messages that either communicated that university students eat a sufficient amount of fruit and vegetable (F&V) or that they do not, on F&V consumption, and whether or not any effects are moderated by student identification. An online 2 (Norm: “Sufficient”/“Insufficient”) × 2 (Identification: “Low”/“High”) experimental design was employed. Infographics containing “sufficient”/“insufficient” F&V intake descriptive norms were presented. An identification manipulation was employed to create “high”/“low” student identifiers. F&V intake intentions were assessed after the manipulations; self-reported F&V intake was reported at 2 days post-intervention. Undergraduate students in the United Kingdom (*N* = 180) reported their intake intentions, of which 112 (62%) completed the behavioral follow-up. Participants were predominantly white female students from Scottish universities, mean age 20.4 (±1.6) years. Baseline mean F&V consumption was high (4.5 ± 2.8). There were no significant main effects of Norm or Identification manipulations on F&V intentions and intake. Significant norm × identification interactions were revealed for fruit intake intentions and vegetable intake at follow-up, indicating half-portion differences (~40 g) between groups. Ironic effects were observed for “high” identifiers, who neither intended to, nor acted in accordance with group norms; “low” student identifiers intended to and followed group norms, whereby the “sufficient”/“low” group intended to consume significantly more fruit portions and consumed more vegetables than the “insufficient”/“low” group. Given the half-portion differences between groups resulting from the norm × identification interactions, future research on a larger sample of young adults with low F&V intake is warranted to further explore the conditions under which moderating effects of identification are observed and the underlying mechanisms.

## Introduction

A robust association exists between fruit and vegetable (F&V) consumption and reduction in all-cause mortality and in the occurrence of several chronic diseases such as cancer and cardiovascular disease (Wang et al., 2014; Aune et al., 2017). The United Kingdom (National Health Service (NHS), 2018) recommends that adults (≥18 years old) should consume at least five portions (5 × 80g) of F&V daily. Despite the introduction of the national “5-a-day” campaign (National Health Service, 2018), the latest surveys show national consumption of F&V falls short of the recommended amount (Rose, 2018; NHS Digital, 2019). The age cohort who consume the least F&V are young adults (16–24 years), who, in England, consume approximately 3.3 portions daily (NHS Digital, 2019), while intake by their Scottish counterparts is lower at 3.2 portions daily (Rose, 2018).

Young adults’ low F&V consumption is concerning as it is the period in which eating habits begin to form, after which resistance to change of established habits increases with age (Gall et al., 2000; Lien et al., 2001). Young adults’ eating behavior is predominantly influenced by peers (Stok et al., 2015; König et al., 2017). Therefore, harnessing social influences may be an effective approach to improving F&V intake (Higgs and Thomas, 2016; Stok et al., 2016).

Social norms are defined as behavioral standards that indicate appropriate and correct behavior (Aronson et al., 1998) and can be used in models as determinants of intentions and behavior. For example, the Theory of Planned Behavior (TPB; Ajzen, 1991) posits that intentions are determined by one’s attitudes, perceived behavioral control, and subjective norms (i.e., one’s norm perceptions) and predicts subsequent behavior from intentions (Ajzen and Madden, 1986). Norms may reflect what the group should be doing, i.e., perceived approval about a behavior (injunctive norms) or what the group is actually doing, i.e., perceived behavior (descriptive norms; Cialdini et al., 1990). Exposure to descriptive norm messages has been found consistently to alter eating behaviors in field experiments (Mollen et al., 2013; Thomas et al., 2017), experimental laboratory studies (Stok et al., 2012, 2014b), systematic reviews, and meta-analyses (Robinson et al., 2014b; Stok et al., 2016 for reviews). Recent research also indicates that descriptive norms are often more successful in increasing F&V intake than conventional messages highlighting the health implications of consuming sufficient F&V (Croker et al., 2009; Mollen et al., 2013; Robinson et al., 2014a).

Prior studies have used non-norm-based messages as a comparator to descriptive norm-based messages. However, de Bruijn et al. (2015) argue that such control messages lack validity. When it comes to “real-life” normative content, it is norms regarding unhealthy behavior—problem behavior—that are most frequently conveyed by mass media and health campaigns (Schultz et al., 2007; Stok et al., 2012; Niederdeppe et al., 2014). de Bruijn et al. (2015) have investigated whether desired descriptive norms were effective when compared with undesired or “problem” descriptive norms on eating intentions (fruit) and behavior, but this small study focused on older adults who may be less sensitive to normative influences than young adults (16–24 years old; Steinberg and Monahan, 2007). Therefore, the impact of descriptive norms highlighting desired behaviors (i.e., sufficient intake) compared with those that focus on problem behaviors (i.e., insufficient intake) on eating intentions and behavior is unclear.

When individuals identify with a group they are more motivated to adhere to in-group norms than out-group norms (Higgs, 2015; Reynolds et al., 2015; Tarrant et al., 2015). Young adulthood is the period throughout which individuals acquire a range of identities (e.g., student) and are motivated by their need to belong (Baumeister and Leary, 1995; Arnett, 2000). Within an eating behavior context, Louis et al. (2007) were the first to investigate the association between group identification strength and perceived eating norms in a longitudinal predictive study. They found high identifiers reported group-congruent intentions, whereas low identifiers did not. However, in a two-week follow-up, identification strength was not found to predict behavior (Louis et al., 2007). Further evidence suggests that the effect of identification strength on the influence of norms on eating behavior is not conclusive (Dempsey et al., 2018), with recent studies suggesting that high identification may result in both norm-divergent behavior (Banas et al., 2016) and convergent behavior (Liu et al., 2019).

The aim of this exploratory research was to investigate whether a descriptive norm message communicating a sufficient F&V intake norm is effective in improving F&V intake intentions and subsequent intake compared with a message communicating an insufficient F&V intake norm. Additionally, we explored whether the influence of the descriptive norm messages depends on the strength of student identification. To examine the effect of identity strength, an identity manipulation was included to categorize participants into distinct “low” and “high” identifier groups.

## Materials and Methods

### Participants and Recruitment

Eligible participants were undergraduate students in the United Kingdom aged 18 years or above, and were recruited *via* social media (e.g., Facebook and Twitter) between April and June 2019. Power analysis using G^*^Power determined a target sample size of 128 participants for ANCOVA that is powered for fixed effects, main effects, and interactions, with alpha set at 0.05 and power at 0.80 (Cohen, 1992) to detect a medium effect size (*f* = 0.25; Erdfelder et al., 2007). This estimate is consistent with previous research demonstrating that studies investigating the effects of eating norms usually detect a small to medium effect size (Robinson et al., 2014b; de Bruijn et al., 2015). Ethical approval was granted by the University Teaching and Research Ethics Committee at the University of St Andrews (MD14242).

### Design

The study employed a randomized, 2×2 between-subjects, pretest/posttest design. The two independent variables were “Descriptive Norm” messages (“sufficient”/“insufficient” F&V intake norm) and “Identification Strength” (“low”/“high”). The study was completed online *via* Qualtrics which, after providing consent, automatically randomized participants in a 1:1 ratio to four groups. The dependent variables were: (a) F&V intake intentions following the norm-based message (see “Part 1”) and (b) self-reported F&V intake at two-day follow-up (see “Part 2”).

### Materials

#### Identification Manipulation

The study involved a between-subjects identification manipulation to expose participants to statements loaded about positive and negative characteristics of student identity (Table 1). Two types of identification manipulations occurred following Greenaway et al.’s (2015) example to create “high” and “low” identifiers. Participants in the “High Identification” group received five moderately positive and five extremely negative student identity-related statements; the “Low Identification” group was presented with five moderately negative and five extremely positive statements (Table 1). Greenaway et al.’s (2015) manipulation posits that the manipulation prompts participants to agree with moderate statements and disagree with the extreme ones. This manipulation has been successfully used by Banas et al. (2016) to create “low” and “high” identifiers in their social norms study. In line with the original manipulation, to ensure participants were aware of the number and valence of selected statements, they were asked to count both the number of negative and positive statements they agreed with. The actual act of counting of the statements participants agree with is the identity primer itself, and the scores were used to indicate identity strength in the analysis. Following this, participants were presented with the norm manipulation (described below).

**Table 1:** Identification manipulation items created following the example of Greenaway et al. (2015).

	“Low” identification	“High” identification
Extreme statements^1^	I identify extremely strongly with other undergraduate university students	I feel no affiliation with other undergraduate students
	It is essential for me that all my friends are undergraduate students	There is no point of doing an undergraduate degree
	I only want to participate in activities with people who are undergraduate students	Being an undergraduate university student opens up no career opportunities in the future
	My undergraduate degree offers me complete control over what I would like to study	Being an undergraduate university student means that all my time is dedicated to studying
	Being a university student means that I can be fully flexible in how I manage my time	There is no sense of community spirit among undergraduate students
Moderate statements^2^	There are some things I do not like about being an undergraduate student	In general, I like being an undergraduate student
	Studying on an undergraduate degree takes up a substantial amount of my free-time	I have friends who are undergraduate students
	I think it is good to have friends outside university	Being a university student provides me opportunities to meet new people
	Studying an undergraduate degree does not always mean that I study about areas that I am interested in	As an undergraduate student, it’s mostly up to me how I manage my own time
	There are some things I do not like about being an undergraduate student	Being an undergraduate university student offers me the opportunity to learn about areas I am interested in

1 *Statements more difficult to agree with*.

2 *Statements easier to agree with*.

#### Norm Manipulation

**Figure 1:**
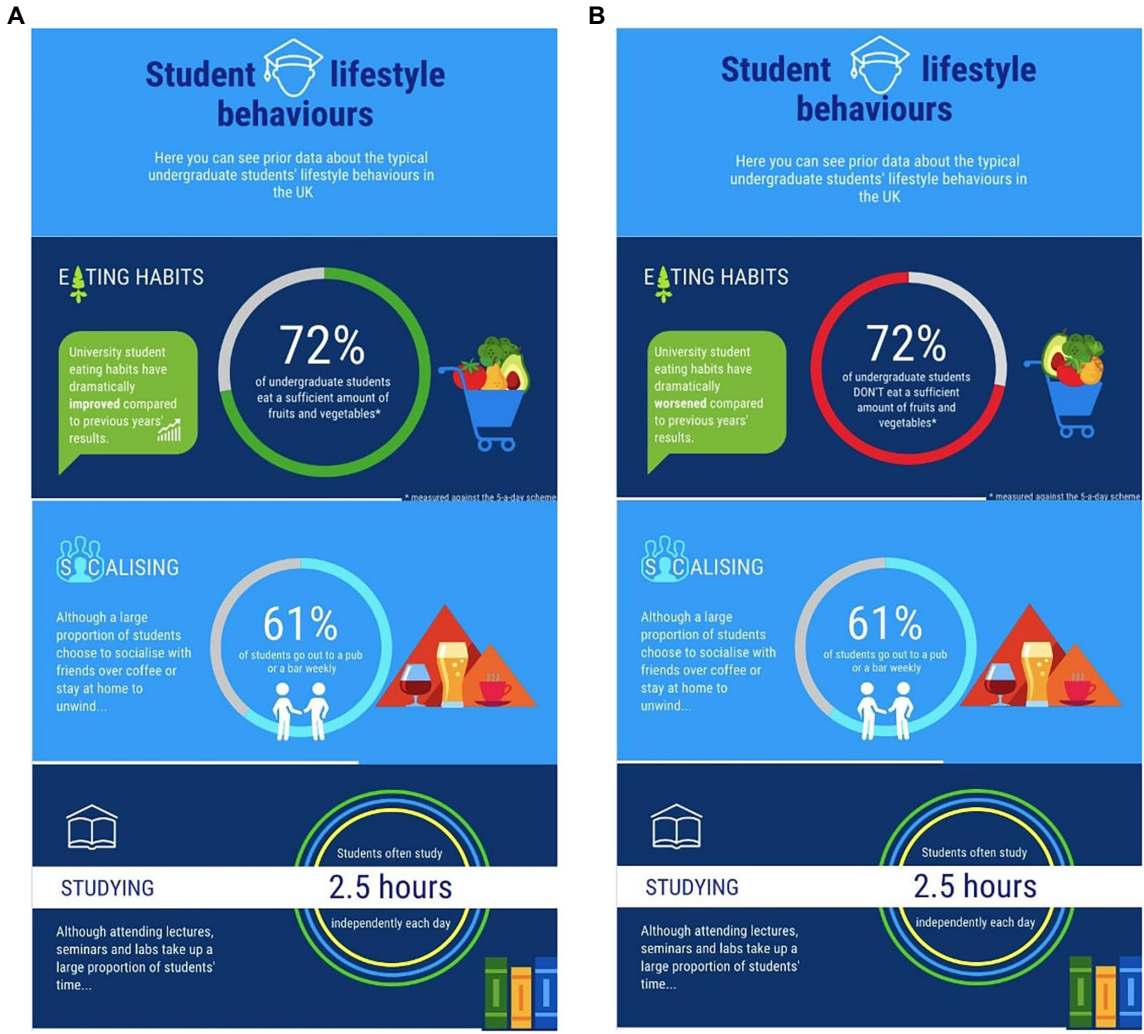
**(A,B)** Infographic containing the “sufficient”/“insufficient” norm.

Following the identification manipulation, participants were asked to rate the clarity of an infographic (Figure 1). Participants were shown one infographic which displayed either a “sufficient” or “insufficient” F&V intake norm alongside additional, unrelated norms (e.g., studying habits) based on a fictitious lifestyle study. Participants were asked to retain the presented information as there was a test afterward, which served as an attention check for their recall of norms.

#### Attention Check

Following the presentation the infographic, participants were asked to recall the percentage of students who eat a “sufficient”/“insufficient” amount of F&V. Answers were accepted to deviate ±10% from the norm presented to them (Banas et al., 2016).

#### Socio-Cognitive Constructs

Attitudes, perceived behavioral control, and intentions to consume sufficient F&V were assessed by items adapted from Ajzen’s (2002) recommendations for creating a scale to measure these constructs in line with previous studies (e.g., Stok et al., 2014b; Table 2). Self-reported F&V intake was assessed by items adapted from Robinson et al. (2014b), which provide an accurate dietary recall over 24 h (Armstrong et al., 2000; Table 2). Guidance on how to determine portion sizes (~80 g) was provided for each question by an image taken from the Scottish Health Survey (Rose, 2018). Fruit and vegetable intake was self-reported separately.

**Table 2:** Measures and corresponding example items, response range, and scoring.

Measures	No. items	Example item	Response range	Scoring	Cronbach’s Alpha^1^
*Socio-cognitive measures*					
Identification as a “sufficient fruit and vegetable eater”	2	“I see myself as someone who eats a sufficient amount of fruit and vegetables.”	Strongly disagree – Strongly agree	1 to 7^3^	0.90
Attitude	4	“Eating 5 portions of fruit and vegetables tomorrow for me would be…”	Unhealthy – Healthy Unpleasant – Pleasant Harmful – Beneficial Unenjoyable – Enjoyable	1 to 7	0.72
Perceived behavior control	4	“For me to eat 5 portions of fruit and vegetables tomorrow would be…”	Impossible – Possible	1 to 7	0.84
Intention to eat 5 portions of fruit and vegetables	4	“I intend to eat at least 5 portions of fruit and vegetables (5x80g) tomorrow…”	Strongly disagree – Strongly agree	1 to 7	0.94
Identification manipulation check	2	“Completing the questions at the beginning of the survey led me to identify as an undergraduate student.”	Strongly disagree – Strongly agree	1 to 7	0.65
*Outcome measures*					
Intended portions to consume the next day	2	“How many portions of vegetables/fruit do you think you will consume tomorrow?”	Number of portions ranging from 0 to 10.5 or more		–
Intake (24h measure)^2^	2	“How many portions of fruit/vegetables did you eat yesterday?”	Number of portions ranging from 0 to 10.5 or more		–

1 *Cronbach’s alpha was employed as a reliability coefficient, for which the desired value was ≥ 0.7 (Nunnally, 1978)*.

2 *This measure was used to assess both baseline and follow-up intake*.

3 *A score of 7 indicates stronger identification/attitudes/perceived behavioral control/intentions; Composite scores were computed for all measures*.

### Procedure

#### Part 1

Participants were invited to complete a 15-min “Lifestyle study” on United Kingdom undergraduate student behaviors. Consenting, eligible participants reported their baseline F&V intake, self-identification as a “sufficient F&V eater,” and socio-cognitive constructs (e.g., attitudes). As the true aim of the study was concealed from participants in an attempt to prevent social desirability bias (Miller et al., 2008), several filler questions were included (e.g., socializing habits), which were not analyzed. Following this, participants received the identification, and then the norm manipulations during the online survey. Demographics [age (year), gender, ethnicity, height (cm), weight (kg), student status (year and country), and dietary requirements] were collected to describe the sample. To match participant responses with the follow-up (see “Part 2” below), participants were guided to create a unique code (see Grube et al., 1989) and provided email addresses.

#### Part 2

Two days after Part 1, upon receipt of the automatic email invitation, participants were asked to self-report the number of F&V portions they consumed the previous day *via* the same 24 h fruit and vegetable online Qualtrics intake form that they answered in Part 1. Participants had the opportunity to enter a prize draw [Amazon voucher (4×£25)]. Upon submission of their answers, a participant debrief form detailed the true aim of the study.

#### Data Analyses

The research questions and the data analysis plan were pre-specified before the data were collected. Differences between the four manipulated groups in baseline F&V intake, demographics, and socio-cognitive constructs were assessed by one-way ANOVAs with group membership as a fixed factor. Manipulation and attention checks were assessed by two-way ANOVAs. Two-by-two ANCOVAs assessed the interaction and main effects of norms and identification manipulations on F&V consumption intentions and behavior (Rausch et al., 2003). Based on previous studies (Robinson et al., 2014a; Stok et al., 2014a), it was decided *a priori* to include attitudes, perceived behavioral control, self-identification as a “sufficient F&V eater,” and baseline intake or intentions as covariates to reduce within-group error variance (Field, 2009). Significant interactions were followed up with Bonferroni-adjusted simple main effects comparisons (Price et al., 2017); significance was determined at *p* < 0.05. Data were analyzed by SPSS v24.

## Results

### Descriptive Statistics

**Figure 2:**
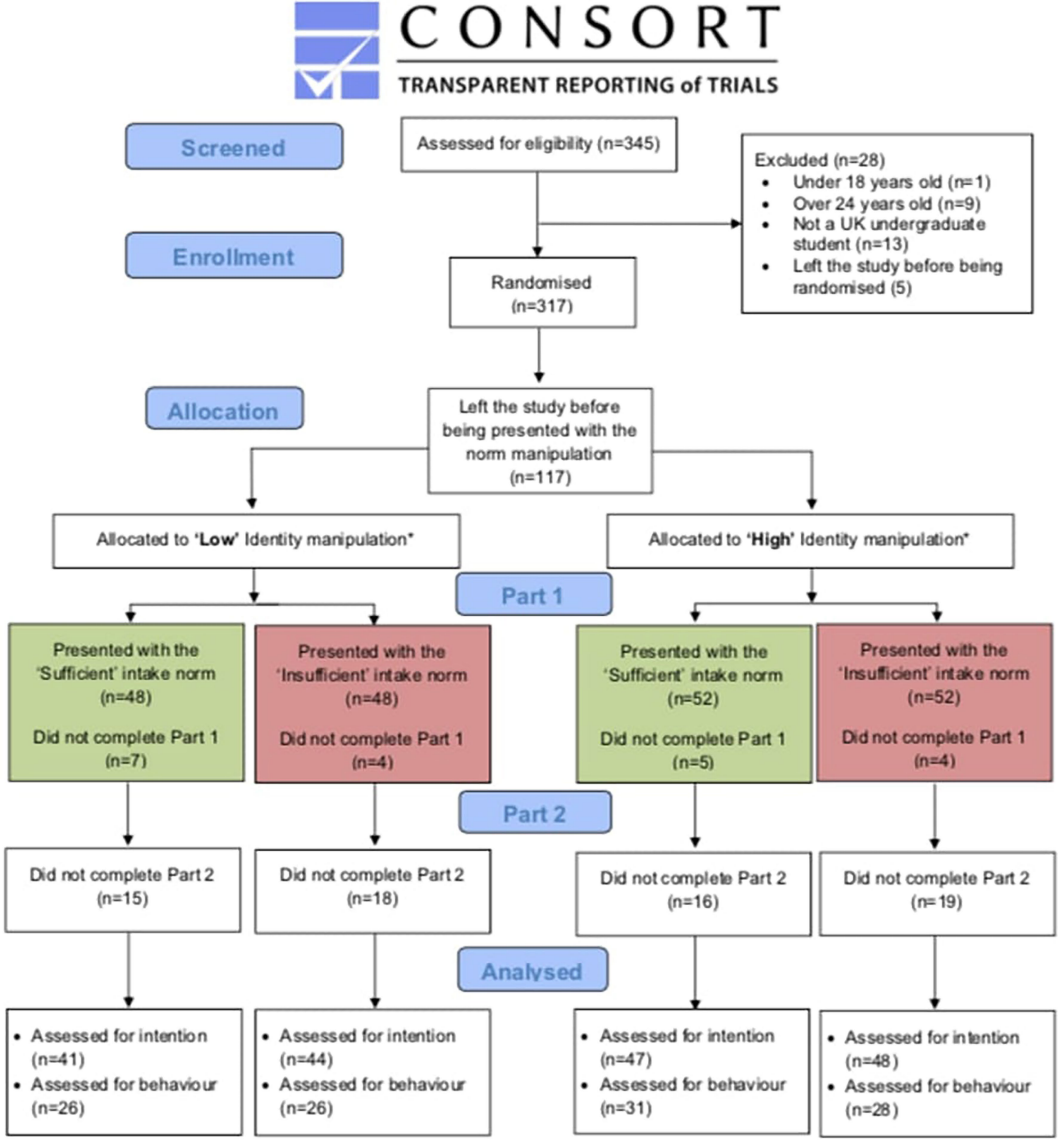
Participant recruitment, allocation, and retention (CONSORT; Schultz et al., 2007). Asterisk indicates that out of the 317 individuals who were allocated to the “low” or “high” identification manipulation, 117 left the study before being presented with the infographics conveying the manipulated norms.

A total of 180 participants completed Part 1 (*M*_Age_ = 20.36 ± 1.64), of which 112 (62.2%) were followed up in Part 2 (Figure 2). Participants not eligible (*n* = 28; e.g., <18–25 > years old, not a student), and those who left the study before (*n* = 117) and after (*n* = 20) being presented with the infographic, were excluded. Sample participants displayed positive attitudes and perceived behavioral control toward consuming 5 portions of F&V a day, as shown by scores above each scale’s mid-point (Table 3). The sample indicated a relatively high baseline F&V consumption, with a mean of 4.50 (SD = 2.86) F&V portions, of which 2.5 (SD = 2.02) and 1.98 (SD = 1.55) mean portions were fruit and vegetables, respectively.

**Table 3:** Means (and standard deviations) of baseline socio-cognitive measures and fruit and vegetable intake.

		“Sufficient” fruit and vegetable intake norm (*n* = 88)		“Insufficient” fruit and vegetable intake norm (*n* = 92)	
Baseline measures		“Low” identification (*n* = 41)	“High” identification (*n* = 47)	“Low” identification (*n* = 44)	“High” identification (*n* = 48)
“Sufficient F&V eater” identification^*^		4.96 (1.50)	4.66 (1.70)	5.11 (1.90)	5.00 (1.81)
Attitude^*^		6.07 (0.93)	5.99 (1.02)	6.04 (1.08)	6.10 (0.85)
Perceived behavioral control^*^		6.02 (1.28)	5.88 (1.17)	6.04 (1.13)	6.02 (1.28)
Baseline fruit and vegetable intake	Fruit	2.09 (1.63)	1.64 (1.31)	1.99 (1.51)	2.24 (2.07)
	Vegetable	2.53 (2.13)	2.50 (1.82)	2.50 (2.04)	2.54 (2.16)
	F&V	4.63 (2.51)	4.13 (2.41)	4.49 (2.71)	4.78 (3.78)

* *Means are based on composite scores, (*N* = 180)*.

Groups did not differ in socio-cognitive constructs, baseline F&V intake, or demographic characteristics (Tables 3, 4; *p* > 0.115). Additionally, the proportionate attrition (Figure 2) was unrelated to condition, demographics, baseline F&V intake, and socio-cognitive constructs (*p* > 0.112). Participants were predominantly White female students from Scottish universities who were normal weight (61%; Table 4; CDC, 2017).

**Table 4:** Participant demographics and breakdown of percentages (*N* = 180).

Characteristics		No. participants (%)	Sufficient F&V intake norm (*n* = 88)		Insufficient F&V intake norm (*n* = 92)	
			“Low” identification (*n* = 41)	“High” identification (*n* = 47)	“Low” identification (*n* = 44)	“High” identification (*n* = 48)
Gender	Female	141 (78.3)	37 (90.2)	36 (76.6)	35 (79.5)	33 (68.8)
	Male	38 (21.1)	4 (9.8)	10 (21.3)	9 (20.5)	15 (31.3)
	Prefer not to say	1 (0.5)	–	1 (2.1)	–	–
Year of study	1st	60 (33.3)	13 (31.7)	18 (38.3)	16 (36.4)	13 (27.1)
	2nd	48 (26.7)	15 (36.6)	12 (25.5)	13 (29.5)	8 (16.7)
	3rd	28 (15.6)	5 (12.2)	5 (10.6)	6 (13.6)	12 (25.0)
	4th	38 (21.1)	6 (14.6)	9 (19.1)	9 (20.5)	14 (29.2)
	5th	6 (3.3)	2 (4.9)	3 (6.4)	-	1 (2.1)
Dietary requirements	Vegetarian/Pescatarian	39 (21.7)	9 (21.6)	6 (12.7)	6 (13.6)	18 (37.5)
	Vegan	12 (6.7)	3 (7.2)	3 (6.4)	4 (9.1)	2 (4.2)
	Allergies/sensitivity/restriction	9 (5.4)	2 (4.8)	3 (6.3)	3 (6.9)	2 (4.2)
	No requirements	120 (66.7)	28 (68.3)	35 (74.5)	31 (70.5)	26 (54.2)
Ethnicity	Asian, Chinese	13 (7.2)	4 (9.7)	2 (4.2)	2 (4.5)	5 (10.5)
	Black	2 (1.1)	–	–	1 (2.3)	1 (2.1)
	Mixed/Other	4 (2.3)	–	1 (2.1)	1 (2.3)	2 (4.2)
	White	159 (88.3)	36 (87.8)	44 (93.6)	40 (90.9)	39 (81.3)
	Prefer not to say	2 (1.1)	1 (2.4)	-	–	1 (2.1)
Country of study^1^	Scotland	166 (92.2)	36 (87.8)	43 (91.5)	42 (95.5)	45 (93.8)
	England	14 (7.8)	5 (12.2)	4 (8.5)	2 (4.5)	3 (6.3)
Body Mass Index (BMI)^2^	Underweight (<18.5)	17 (9.4)	5 (12.2)	3 (6.4)	4 (9.1)	5 (10.4)
	Normal (18.5–24.9)	110 (61.1)	26 (63.4)	31 (66.0)	28 (63.6)	25 (52.1)
	Overweight (25.0–29.9)	32 (17.8)	6 (14.6)	8 (17.0)	6 (13.6)	12 (25.0)
	Obese (>30.0)	8 (4.4)	3 (7.3)	1 (2.1)	3 (6.8)	1 (2.1)
	Not available	13 (6.7)	1 (2.4)	4 (8.5)	3 (6.8)	5 (10.4)

1 *There were no participants who studied at Welsh or Northern Irish universities*.

2 *Calculated based on self-reported height (cm) and weight (kg) and classified according to the Centre for Disease Control and Prevention (CDC, 2017) cut-off points for adults aged ≥18 years*.

### Identification Manipulation Check

ANOVA revealed a non-significant difference between the “low” (4.73 ± 1.34) and “high” (5.07 ± 1.34) identification conditions, *F*(1,179) = 2.97, *p* = 0.086, indicating the manipulation was not fully successful.

### Attention Check

A total of 76.1% of participants recalled the descriptive norms displayed by the infographics correctly. Participants rated the infographics as easy to understand and well-presented. There was a significant difference in descriptive norm recall between conditions, with a larger percentage of correct recalls in the “insufficient” (84%) than the “sufficient” condition (68%), *F*(1,178) = 6.09, *p* = 0.015, $\eta_{p}^{2}$ = 0.03.

### Intentions

#### Intention to Eat >5 F&V Portions (Part 1)

**Figure 3:**
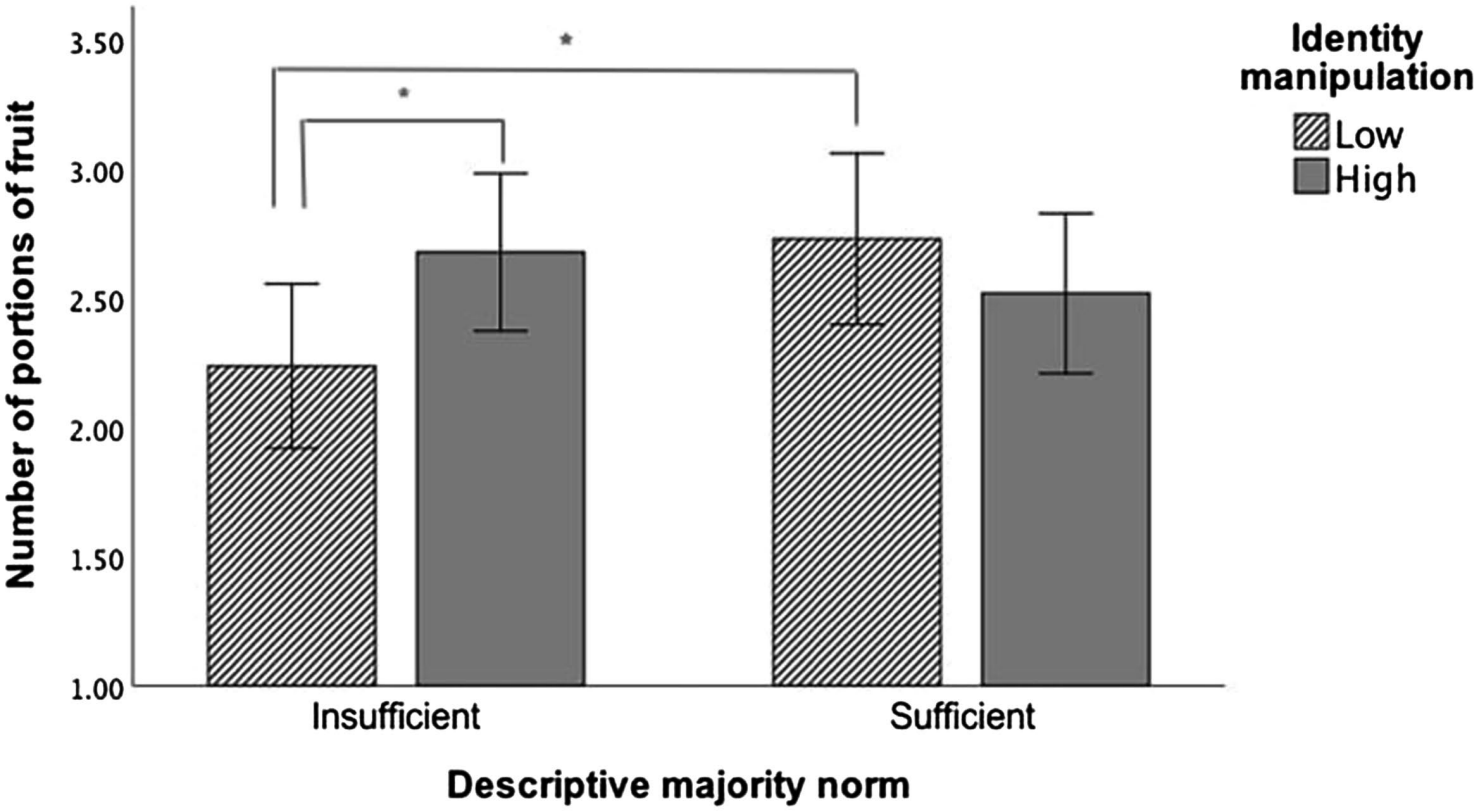
Bar graph illustrating the cross-over interaction of the Norm (“sufficient”/“insufficient”) and Identification (“low”/“high”) manipulations on the number of portions of fruit participants intended to consume the following day. The means are adjusted for baseline intake, attitudes, perceived behavioral control, and identification as a “sufficient fruit and vegetable eater.” Error bars display 95% confidence intervals. Asterisk indicates significant difference at ^*^p < 0.05. (*N* = 180).

ANCOVA revealed no main effects of norms or identification on fruit intake intentions. The norm by identification manipulation interaction was significant, which generated a small effect size (Table 5). Simple main effects analysis revealed that when presented with “insufficient” norms, participants in the “high” identification group reported intentions to eat approximately half a portion more fruit (Mean difference_adjusted_ = 0.44, *p* = 0.05) than participants in the “low” identification manipulation group (Figure 3). Additionally, “low” identifiers in the “insufficient” condition intended to consume significantly fewer portions (Mean difference_adjusted_ = −0.49, *p* = 0.036) than participants in the “sufficient” condition (Figure 3). No main effects (norm or identification) nor interactions were found for vegetable intake intentions (Table 5). No main effects nor interactions were found for “overall intentions” to consume ≥5 F&V portions the next day (Table 5).

**Table 5:** ANCOVA table for fruit and vegetable intake intentions (Part 1).

Independen*t* variables	*F*(1,172)			*p*			$\eta_{p}^{2}$		
	Fruit	Vegetable	Overall intentio*n*^2^	Fruit	Vegetable	Overall intentio*n*^2^	Fruit	Vegetable	Overall intentio*n*^2^
Norm manipulation	1.09	0.97	0.03	0.299	0.326	0.862	0.01	0.01	0.00
Identification manipulation	0.52	0.85	1.02	0.474	0.357	0.314	0.00	0.01	0.01
Norm × Identification manipulation	4.11	2.25	0.10	0.044^*^	0.136	0.757	0.02	0.01	0.00
Covariates									
Baseline intake^1^	119.59	102.02	9.48	0.001^*^	0.001^*^	0.002^*^	0.41	0.37	0.05
Attitude	2.00	5.38	38.35	0.159	0.020^*^	0.001^*^	0.01	0.03	0.18
Perceived behavioral control	1.44	0.15	4.92	0.233	0.700	0.028^*^	0.01	0.00	0.03
“Sufficient fruit and vegetable eater” identification	0.22	5.95	44.69	0.642	0.016^*^	0.001^*^	0.00	0.03	0.21

* *Significant at *p* < 0.05*.

1 *Baseline intake refers to corresponding food type (fruit/vegetable/fruit and vegetable)*.

2 *Overall intentions refer to intention to eat 5 or more portions of fruit and vegetables the next day; (*N* = 180)*.

#### Intake: Number of F&V Portions Consumed (Part 2)

**Figure 4:**
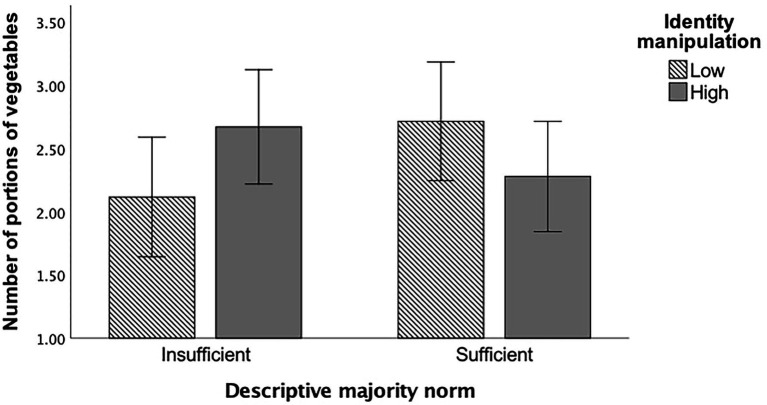
Bar graph illustrating the cross-over interaction of the Norm (“sufficient”/“insufficient”) and Identification (“low”/“high”) manipulations on the number of vegetable portions participants consumed at follow-up. The means are adjusted for attitudes, perceived behavioral control, intentions, and identification as a “sufficient fruit and vegetable eater” as covariates. Error bars represent 95% confidence intervals. (*N* = 111).

Participants self-reported their F&V intake in the two-day follow-up (Table 6). No main effects (norm and identification) nor interactions were found for self-reported fruit intake two days post-intervention (Table 7). No significant main effects were revealed for norm nor identification on vegetable intake (Table 7). The norm by identification manipulation interaction was significant (*p* = 0.034), which generated a small to medium effect size (Table 7; Figure 4). Although it was not significant, simple main effects analysis revealed that upon receiving the “insufficient” intake norm, participants in the “high” identification consumed approximately half a portion more vegetables (Mean difference_adjusted_ = 0.55, *p* = 0.095) than participants in the “low” condition. Participants under the “high” identification manipulation who received the “sufficient” intake norm consumed fewer portions (Mean difference_adjusted_ = −0.44, *p* = 0.179) than participants receiving the “low” identity manipulation. Additionally, a non-significant, half-portion difference (Mean difference_adjusted_ = 0.60; *p* = 0.079) was detected between the two “low” identifier groups, with those in the “sufficient” norm condition consuming more vegetables.

**Table 6:** Means (and Standard Deviations) for fruit and vegetable intake at two-day follow-up (Part 2).

Follow-up intake	“Sufficient” fruit and vegetable intake norm (*n* = 57)		“Insufficient” fruit and vegetable intake norm (*n* = 55)	
	“Low” Identification (*n* = 26)	“High” Identification (*n* = 31)	“Low” Identification (*n* = 26)	“High” Identification (*n* = 29)
Fruit	2.52 (1.84)	1.65 (1.23)	2.08 (1.63)	2.43 (1.39)
Vegetable	2.69 (1.85)	1.87 (1.23)	2.40 (1.60)	2.87 (1.78)
F&V	5.21 (3.30)	3.52 (1.98)	4.48 (2.76)	5.12 (2.85)

**Table 7:** ANCOVA table for fruit and vegetable intake at two-day follow-up (Part 2).

Independent variables	*F* (1, 103)		*p*		$\eta_{p}^{2}$	
	Fruit	Vegetable	Fruit	Vegetable	Fruit	Vegetable
Type of Norm	0.20	0.19	0.776	0.655	0.00	0.00
Identification manipulation	0.11	0.07	0.918	0.739	0.00	0.00
Type of Norm × Identification manipulation	0.35	4.606	0.558	0.034^*^	0.00	0.04
Covariates						
Portions intended to consume^1^	65.98	50.24	0.001^*^	0.001^*^	0.39	0.33
Attitude	3.34	0.01	0.071^*^	0.908	0.03	0.00
Perceived behavioral control	0.52	1.97	0.472	0.163	0.01	0.01
“Sufficient fruit and vegetable eater” identification	1.26	0.00	0.218^*^	0.264	0.01	0.00

* *Significant at *p* < 0.05*.

1 *Corresponding food type (Fruit/Vegetable); (*N* = 112)*.

## Discussion

This study compared the effect of a descriptive norm message communicating the “sufficient” F&V intake of in-group members with an “insufficient” F&V intake message, on immediate F&V intake intentions and subsequent intake at a two-day follow-up. Whether the impact of descriptive norms was dependent on student identification strength was also investigated by employing a manipulation to categorize students as “low”/“high” identifiers. The manipulation was not fully successful in creating distinct “low”/“high” identifiers, and participants in the “low” identification group displayed relatively high identification, which is frequently observed in studies (Stok et al., 2012; Banas et al., 2016; Liu and Higgs, 2019). We found that participants in the “low” identification group intended to and acted norm-congruently, while participants in the “high” identification group intended to and acted against the presented norms.

Some social norms research asserts that higher identification predicts norm-congruent behavior (Louis et al., 2007; Stok et al., 2012, 2014a). For example, in a similar study, Liu et al. (2019) investigated the relationship between descriptive norms and identification strength on participants’ F&V intake. The researchers primed one group’s student identity and showed participants a flyer or a poster either communicating descriptive norms about most students consuming over 5 portions of F&V daily or a health message conveying the benefits of eating 5-a-day. They found the primed group consumed 40 g more F&V from a buffet than the non-primed group. In contrast, the present data show that only the “low” identification group participants’ fruit intake intentions and vegetable intake were norm-congruent. Several explanations may be attributable to the differences in the current findings and Liu et al.’s (2019) experiment. Crucially, Liu et al.’s (2019) sample consumed 2 F&V portions at baseline, whereas our sample reported 4.5 portions. Previous studies have indicated substantial differences in the effect of norms on “high” and “low” F&V consumers, with “low” consumers being more prone to match norms (Schultz et al., 2007; Robinson and Higgs, 2012; Robinson et al., 2014a; Verkooijen et al., 2015). Therefore, the contrasting findings could be attributed to baseline F&V intake. Additionally, the difference between the identity manipulations used by Liu et al. (2019) and in the present study may also account for the observed contradictory results.

The present data also suggest that participants in the “high” identification group actually diverged from norms. This manifested in the present study in two ways. Firstly, participants in the “high” identification group intended to eat more fruit and increased their vegetable intake upon receiving the “insufficient” descriptive norm, although this was not significant. This may be explained by their desire not to be associated with a group which has unfavorable norms (Berger and Heath, 2007; Berger and Rand, 2008), leading to a compensatory behavior. Secondly, participants in the “high” identification group intended to eat fewer fruit portions and (non-significantly) decreased their vegetable intake by half a portion upon receiving the “sufficient” descriptive norm. The finding corroborates Banas et al. (2016), who demonstrated that “high” identifiers chose calorific food items from an online menu when presented with “healthy” descriptive norms, indicating the presence of ironic effects. Banas et al. (2016) suggested that observed ironic effects could be explained by vicarious licensing. Vicarious licensing posits when high identifiers perceive their in-group members making progress in achieving a goal (e.g., eating healthily), they may give an individual license to themselves (e.g., choose unhealthy food; Kouchaki, 2011). This ironic effect has primarily been associated with hedonic consumption, where one is offered an alternative choice (Wilcox et al., 2009; De Witt Huberts et al., 2012). However, as the current study did not investigate vicarious licensing, nor offer an alternative choice, a definitive conclusion cannot be drawn as to whether this is the underlying mechanism for the findings. Taken together, the data suggest that understanding of the moderating effects of identification on responses to eating norms requires further investigation.

In the present study, an approximate half-portion (~ 40 g) difference (non-significant) was consistently observed between descriptive norm conditions, which is noteworthy, given that long-term school-based dietary interventions for children (5–12 years) can only demonstrate an increase in F&V intake by an average of one-quarter to one-third of a portion (~20–30 g; Evans et al., 2012). The half-portion difference is clinically relevant given the dose-related relationship between F&V intake and diseases such as cardiovascular disease and cancer (Aune et al., 2017), and evidence indicating that each additional serving of fruit or vegetable a day is associated with 5–6% reduced risk of all-cause mortality (Wang et al., 2014).

It is important to note that as the manipulation was not fully successful to create distinct “low”/“high” identifiers, participants in the “low” identification group displayed relatively high identification, which is frequently observed in studies (Stok et al., 2012; Banas et al., 2016; Liu and Higgs, 2019). Consequently, the two identification groups could be regarded as “identifiers” and “extreme identifiers,” whereby “identifiers” perceive descriptive norms relevant, and thus act norm-congruently, a well-documented finding (Cruwys et al., 2012; Stok et al., 2012; Reynolds et al., 2015). Our findings showed that the intention and consumption of “identifiers” were poorer in the “insufficient” condition, which may be attributed to the “backlash” effect (Cialdini, 2003), which is an undesired behavioral outcome following exposure to undesired norms conveying problem behaviors about one’s in-group.

Our findings in relation to the “sufficient”/“desired” descriptive majority norms compared with “problem”/“insufficient” norms on intention and behavior show that when identity is not taken into account, there were no differences in their impact. These findings support the only similar investigation conducted to date by de Bruijn et al. (2015), who found desired descriptive norms had no effect on fruit intake intentions and intake when compared with undesired, “problem norm” content. These findings are broadly consistent with available field research on drinking behavior (Foxcroft et al., 2015). However, an explanation for the non-significant differences between descriptive norms may also lie in participants’ norm recall rates. Participants in the “insufficient” condition recalled norms more successfully than those in the “sufficient” condition, suggesting the bogus “sufficient” norm was perhaps perceived as inaccurate. In support of this suggestion, research shows students are generally perceived to be “unhealthy” (Tarrant and Butler, 2011) with students often overestimating peer’s poor health behaviors (Neighbors et al., 2006).

### Strengths

A strength of this investigation is the norm-conveying infographics—regarded as well presented and clear by participants—which were designed to resemble content encountered in daily life. Therefore, the infographics are ecologically valid and can be employed in future research. Furthermore, the concealment of true study objectives during recruitment and the absence of the experimenter throughout data collection lessened the likelihood of social desirability bias, a bias commonly experienced in eating behavior research (Steim and Nemeroff, 1995; Nix and Wengreen, 2017). Overall, the investigation contributes to the limited experimental social norm studies exploring healthy eating and employing a follow-up self-reported intake measure, as opposed to immediate food choice measures or intention only (Robinson, 2015; Stok et al., 2018).

### Limitations and Recommendations for Future Research

A limitation of this study relates to the identification manipulation which was not fully successful in creating distinct “low”/“high” identifiers, thereby limiting variability to detect a moderating role for identification on descriptive norm messages. Future research should aim to improve the manipulation to verify the direction of the interaction of descriptive norms and identification strength. Additionally, the analysis of intake was underpowered due to attrition and is acknowledged as a limitation. Furthermore, the norms were fictitious and norm recall rates were significantly different between the norm conditions. It is possible that the insufficient F&V intake norm manipulation seemed more credible compared to the sufficient norm manipulation to the participants. Future studies may test pre-existing norm perceptions and/or assess whether the norms are regarded as credible. An unexpected finding was the discrepancy observed between F&V intake intentions and behavior. Although measuring intentions is appropriate in predicting behavior (Kellar and Abraham, 2005; Ickes and Sharma, 2011), intentions do not necessarily manifest (Lien et al., 2001; Sniehotta et al., 2005) resulting in an intention-behavior gap.

The “lifestyle study” ostensibly attracted health-motivated participants, potentially leading to selection bias. This may explain why participants identified as “sufficient F&V eaters” and displayed relatively strong attitudes, perceived behavioral control, and intentions to eat 5-a-day. Additionally, the sample’s BMI distribution (61% healthy BMI) is not representative of adults in Scotland, as recent evidence indicates that prevalence of overweight (including obesity) is 65% for this cohort (Bardsley, 2018). Furthermore, asking participants to self-report their sufficient F&V eater identity may have had a priming effect that impacted the results.

As participants’ self-reported baseline consumption was at 4.5 F&V portions, which conforms approximately to the 5-a-day norm presented, it is plausible that a ceiling effect was observed. The sample’s baseline consumption is substantially higher than intake levels reported in national surveys of young adults’, and cross-sectional investigations of undergraduate students’ eating practices (Tanton et al., 2015; Rose, 2018; Sprake et al., 2018). This may be due to the high proportion of vegetarian/vegan participants at 28.4% in the sample, who typically eat more F&V than meat-eaters (Walsh et al., 2017). Overall, the external validity of the sample is thus limited, which is furthered by the predominant participation of white, female students studying in Scotland. Future research obtaining larger and demographically diverse samples displaying the nationally observed low F&V consumption is warranted.

### Practical Implications

The finding that participants in the “low” identification group intended to and consumed fewer portions when presented with “insufficient” descriptive norms tentatively suggests that this kind of normative content may instigate unwanted outcomes (i.e., “backlash effect”), and therefore, conveying descriptive norms about problem behaviors in health promotion material should be cautioned. Additionally, the present findings add to the disagreement in the literature regarding the direction of the norm×identification interaction due to potential ironic effects for participants in the “high” identification group’ intentions and behavior. Hence, these findings warrant further investigations of the underlying mechanisms, such as vicarious licensing, to offer a solution for harnessing the benefits of in-group identification in health promotion.

## Conclusion

Although descriptive norms offer a cost-effective and simple approach to improve F&V intake intentions and behavior, and are successful when compared with no-norm controls and health messages (Robinson et al., 2014a), their effectiveness has not yet been demonstrated compared with undesired normative content in an eating behavior context. However, descriptive norms influenced fruit intake intentions and vegetable intake when investigated for their interaction with the identification manipulation, with participants in the “low” identification group acting norm-congruently, and participants in the “high” identification group diverging from the presented norms. The latter potentially suggests the ironic effects of high identification on behavior. Whether the findings generalize to other health behavior contexts, and to the general young adult population who would benefit from F&V intake improvement, remains subject to further investigation.

## Data Availability Statement

The raw data supporting the conclusions of this article will be made available by the authors, without undue reservation.

## Ethics Statement

The studies involving human participants were reviewed and approved by the University Teaching and Research Ethics Committee at the University of St Andrews (MD14242). The participants provided their written informed consent to participate in this study.

## Author Contributions

All authors listed have made a substantial, direct, and intellectual contribution to the work and approved it for publication.

## Funding

The funding for open access was supported by the University of St Andrews.

## Conflict of Interest

The authors declare that the research was conducted in the absence of any commercial or financial relationships that could be construed as a potential conflict of interest.

## Publisher’s Note

All claims expressed in this article are solely those of the authors and do not necessarily represent those of their affiliated organizations, or those of the publisher, the editors and the reviewers. Any product that may be evaluated in this article, or claim that may be made by its manufacturer, is not guaranteed or endorsed by the publisher.
